# Understanding Training Load as Exposure and Dose

**DOI:** 10.1007/s40279-023-01833-0

**Published:** 2023-04-06

**Authors:** Franco M. Impellizzeri, Ian Shrier, Shaun J. McLaren, Aaron J. Coutts, Alan McCall, Katie Slattery, Annie C. Jeffries, Judd T. Kalkhoven

**Affiliations:** 1 Faculty of Health, Human Performance Research Centre, School of Sport, Exercise and Rehabilitation, University of Technology Sydney, Sydney, NSW 2007 Australia; 2 Centre for Clinical Epidemiology, Lady Davis Institute, Jewish General Hospital, McGill University, Montreal, QC Canada; 3 Newcastle Falcons Rugby Club, Newcastle upon Tyne, UK; 4 Department of Sport and Exercise Sciences, Durham University, Durham, UK; 5 Arsenal Performance and Research Team, Arsenal Football Club, London, UK; 6 College of Engineering, Science and Environment, School of Environmental & Life Sciences, The University of Newcastle, Newcastle, NSW Australia

## Abstract

Various terms used in sport and exercise science, and medicine, are derived from other fields such as epidemiology, pharmacology and causal inference. Conceptual and nomological frameworks have described training load as a multidimensional construct manifested by two causally related subdimensions: external and internal training load. In this article, we explain how the concepts of training load and its subdimensions can be aligned to classifications used in occupational medicine and epidemiology, where exposure can also be differentiated into external and internal dose. The meanings of terms used in epidemiology such as exposure, external dose, internal dose and dose–response are therefore explored from a causal perspective and their underlying concepts are contextualised to the physical training process. We also explain how these concepts can assist in the validation process of training load measures. Specifically, to optimise training (i.e. within a causal context), a measure of exposure should be reflective of the mediating mechanisms of the primary outcome. Additionally, understanding the difference between intermediate and surrogate outcomes allows for the correct investigation of the effects of exposure measures and their interpretation in research and applied settings. Finally, whilst the dose–response relationship can provide evidence of the validity of a measure, conceptual and computational differentiation between causal (explanatory) and non-causal (descriptive and predictive) dose–response relationships is needed. Regardless of how sophisticated or “advanced” a training load measure (and metric) appears, in a causal context, if it cannot be connected to a plausible mediator of a relevant response (outcome), it is likely of little use in practice to support and optimise the training process.

**Supplementary Information:**

The online version contains supplementary material available at 10.1007/s40279-023-01833-0.

## Key Points

**Table:** 

Terms that are used in sports science and medicine, such as exposure, dose and response, are derived from other scientific disciplines.
Training load-related classification and framework are consistent with definitions and concepts used in other areas of science where causal inference is utilised, such as epidemiology, pharmacology and clinical research.
The term load is the equivalent of dose (amount of something) but also conveys the idea of demands challenging the organism.
External and internal training load conceptually differentiate what athletes do (external dose) from the psychobiological responses caused by these activities (internal dose).
As in other fields, any measure of training load should provide information (e.g. about mediating mechanisms) to support the planning and execution of a training programme aimed at improving characteristics that are causally linked to performance (or to any outcome of interest).

## Introduction

Various terms used in sport and exercise science, and medicine, are derived from epidemiology and other biomedical sciences, such as pharmacology. For example, many studies commonly utilise terms such as exposure and dose when referring to training and exercise [1–4]. Within the various areas of epidemiology and pharmacology, exposure and dose have been conceptualised and classified in many ways. Understanding these concepts is important for the selection of appropriate measures of exposure and dose, as well as the exploration of other related concepts such as the dose–response relationship. In this article, the meanings of various common terms within the physical training vernacular are reconciled with similar classifications and conceptualisations used in epidemiology and pharmacology.

### Article Scope

The purpose of this article is to acknowledge recent discussions regarding the use of training load-related terminology and concepts including the interpretation of training load as dose [5–9], as well as providing further clarification of the meaning and role of these terms within the training process framework by drawing parallels with epidemiology. Training can have various effects, and understanding what measures of exposure to use and the appropriate metrics (i.e. cumulative, volume, average and peak intensity, overall pattern) depends on the goal of the training, the responses of interest (primary outcomes) and the relevant aspects of training that the coaches, practitioners or researchers are interested in. Because of the numerous outcomes of interest (performance or health related such as athletic injuries), each with their own set of mediators (mechanisms), there *cannot* be a single measure that reflects all the mediators of the various outcomes.

In addition, most measures of training load attempt to quantify latent variables that cannot be directly observed (and hence “objectively” measured) but can only be estimated through their effect on observed variables. We therefore also explain how key concepts presented in epidemiology can assist in the identification and validation of measures of training load when the aim is to optimise the training process. In this context, “optimise” (and optimisation) specifically refers to the manipulation or variation of physical training parameters to obtain better outcomes, which implies (interventionist) causation. For this reason, and because we hope sports science and medicine will continue to utilise and/or implement advancements in the area of causal inferences made in other fields, we use the causal inference perspective and counterfactual terminology, which is consistent with the causality principles developed over the last 40 years in areas such as computer science [10], economics [11], statistics [12] and epidemiology [13].

In the structural causal model framework, we rely on directed acyclic graphs (one type of causal diagram) to display *some* assumptions about the underlying causal relationships. We also addressed the topic from a macro- and not micro-level perspective. We direct the readers interested in better understanding basic terminology relating to casual inferences and diagrams to other papers [14, 15]. As this article focused on the concepts of exposure and dose, we will not elaborate on the implicit causal inference principles and the meaning of counterfactual terminology. Issues such as time-varying exposure/dose and statistical models represent more complex topics that are beyond the scope of this article, but the principles we discuss are applicable in those contexts as well. Furthermore, in this article, we did not adopt a dynamic system perspective, which is a different philosophical and methodological approach to causality, causes and interactions [16–20]. However, we added a supplementary document to clarify some of these concepts and the different use of some terms (e.g. interaction and causes) under different philosophical approaches to causation. This was included to avoid misunderstandings and to acknowledge there are different approaches.

## Exposure and Dose

Epidemiologists use different definitions of exposure (descriptive and operational) in different contexts. For the current article, we refer to the exposure definitions presented in a reference dictionary of epidemiology (Table 1) [21]. At least two of these definitions can be contextualised to the physical training process proposed for sport and exercise science [22, 23].

**Table 1:** Definitions of exposure [21]

Def 1	The variable whose causal effect is to be estimated
Def 2	Proximity and/or contact with a source of a disease agent in such a manner that effective transmission of the agent or harmful effects of the agent may occur ﻿
Def 3	The amount of a factor to which a group or individual was exposed, ﻿sometimes contrasted with dose, the amount that enters or interacts with the organism
Def 4	The process by which an agent comes into contact with a person or animal in such a way that the person or animal may develop the relevant outcome, such as a disease

*Def* definition

As per definition 1 (Table 1), exposure is a variable whose causal effect is to be estimated, regardless of whether this variable is external or internal to the body. In other words, exposure refers to any variable of interest that might cause the outcome [24]. In the causal framework, a cause is defined as a variable that results in the outcome occurring if it is present, and the outcome not occurring if it is absent. In most cases, a variable only causes the outcome if some other variables are present; i.e. there is a biological interaction between them. The entire set of variables together is called a sufficient causal set, and each individual variable is called a component cause [25–27]. A more detailed explanation is provided in the Electronic Supplementary Material (ESM).

Causal variables can include behaviours, treatments, interventions, hazards and traits [28, 29], as well as variables typically used in exercise physiology such as heart rate, blood pressure and serum glucose. In sports and exercise, we commonly want to estimate the causal effect of training on sporting performance or health-related outcomes. Accordingly, training load has been defined as an input variable that is manipulated to elicit the desired training response, which identifies training as a causal variable (as per definition 1) [30]. Therefore, in the physical training context, “training” is the variable of interest, i.e. the exposure. However, this definition does not specify the scale of the variable [31]. By adding the term “load” (which indicates an amount) to “training”, it is implied that we are generally referring to a continuous or at least ordinal, instead of categorical (e.g. yes/no) variable. It follows that, when applying definition 1 to physical training, “training load” acts as a generic term referring to the amount of the exposure variable irrespective of whether this exposure is external or internal to the body. However, within the physical training process framework, training load has been sub-categorised into internal and external (training) load [28, 29], the implications of which will be discussed in more detail in the following sections.

Unlike definition 1, definition 3 (Table 1) more explicitly states that exposure is considered to be a continuous (or ordinal) variable: “the *amount* of a factor to which individuals are exposed” [21]. Exercise, physical activity and diet, and even events such as heading the ball in football or physiological/biomechanical constructs, are factors acting on the body, i.e. factors to which individuals are “exposed” [32, 33]. This definition is arguably more consistent with the training load concept used in sports science as the term “training” acts as a qualifier identifying the factor of interest to which individuals are exposed, and the term “load” indicates the amount of this factor [22, 23, 34]. As per definition 1, when applying definition 3 to physical training, “training load” includes any measure of load without reference to whether it is external or internal to the body, i.e. whether it is a measure of external or internal load.

Other definitions presented in Table 1 along with alternative definitions that can commonly be found in the literature and relevant textbooks, often use the term “agent” interchangeably with “factor” and “variable”. “Agent” is a generic term used to indicate substances (e.g. pollution and drugs) but can be extended to any other attributes. For example, exposure has been defined by Cordier and Stewart [35] “as a contact of an individual with an agent through any medium or environment.” These authors clarified that exposure can be a chemical, biological, physical or societal agent in the external environment, or characteristics of an individual (including weight and physical activity), susceptibility, exercise, diet and any other external or internal agent [35]. Others extend the definition to include any activity or action that can be directly or indirectly measured [21]. Accordingly, the epidemiological concepts and definitions of agents (or factors) have already been legitimately applied and adapted to physical activity and exercise.

Within Table 1, definition 3 introduces another important concept: dose. Exposure and dose are often used interchangeably or even combined (e.g. dose of exposure) depending on the scientific context. However, in some areas such as environmental and occupational epidemiology, the concept of dose is differentiated from exposure. Definition 3 specifically states that exposure is sometimes contrasted with dose, where dose is defined as “the *amount* of a substance [agent or factor] that enters or interacts with the organism.” [21] The reason for this contrast is that it is also common to think of exposure as an “agent in the external environment” [36]. The key aspect of these dose definitions is that they refer to the actual stimulus “that enters or interacts with the body”, which is what triggers biological adaptations. In some contexts, causal effects occur when a dose exceeds a certain threshold. In other contexts, causal effects may increase as the dose increases, or be dependent on the total amount of exposure.

## External and Internal Dose

**Fig. 1:**
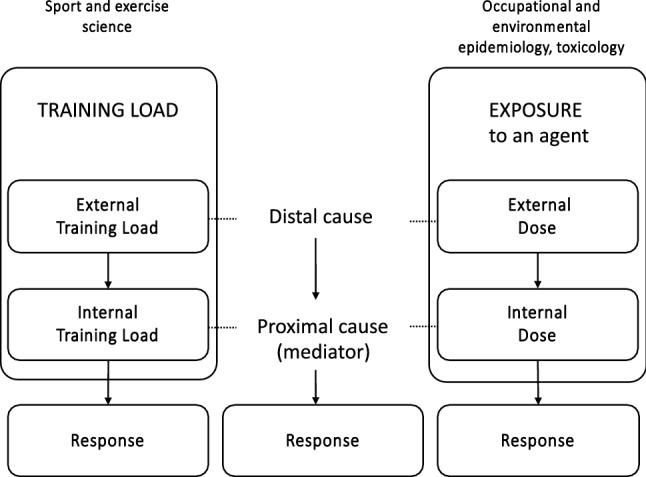
Diagram depicting the parallels exhibited between terms used in sports science and certain areas of epidemiology and pharmacology. The left and right diagrams represent classifications where the training load and exposure include two different but related subdimensions (external and internal load and dose). The central section provides the role of the two subcomponents in the causal pathway leading to a response. The internal dose and load are more proximal causes of the outcome of interest, i.e. they are mediators of (or mechanisms for) the effect of the external dose/load on the outcome

The previous definitions for exposure and dose can be conceptually applied to physical training in a sport and exercise context. However, these definitions do not clearly reflect the three elements of the training process framework, which include training load, external (training) load and internal (training) load (Fig. 1) [23].

In the physical training process, training load is a multidimensional construct that is manifested by the two causally related sub-dimensions: external and internal load (Fig. 1). Within (environmental and occupational) epidemiology and pharmacology (e.g. toxicology), there is a parallel classification of exposure, where the generic term “exposure” encompasses two causally related subdimensions. In this classification, internal dose (the amount individuals internally absorb, i.e. systemically available) [37–39] is a mediator of the effect of external dose (the amount individuals are physically exposed to) on the outcome/response (Fig. 1) [38]. The parallel between these classifications is therefore quite straightforward. Training load represents the exposure (overarching construct) where its two causally related sub-dimensions, external and internal training load (manifestations of the multidimensional construct), act as conceptual equivalents to external and internal dose. Training load/exposure can therefore be quantified by measures reflecting its two manifestations; i.e. by using measures reflecting external and/or internal (training) load/dose.

Training load is a relatively simple bi-dimensional construct where the causal relationship of its sub-dimension is intuitive and coherent with available classifications used in other fields. A conceptual framework showing how internal and external training loads are related and may interact within the training process (together with other contextual and individual factors) has been previously presented [23]. This structure is widespread in sports and exercise science and can be helpful in both practice and research settings to (1) understand how and what components of the training process we can measure, (2) provide a reference framework for the validation of the measures of external and internal load and (3) help to understand the role of specific measures (and the characteristic they reflect) in the causal pathway leading to the response/outcome of interest. The external dose is therefore the amount of training (agent/factor) to which individuals are exposed, and is quantified using measures reflecting the amount of physical training (activities and actions) performed by athletes [22, 23, 34]. The internal dose is the internal (to the body) training load that an individual experiences to cope with the demands imposed by the external load, and is quantified using measures reflecting the psycho-physiological stress during the training/exercise, or any other internal load measures.

In epidemiology and pharmacology, external dose is expected to cause different internal doses between and within individuals as internal dose is influenced by factors such as genetics, metabolism, susceptibility and changes in states [32, 40]. For the same reasons, within the physical training process, a given external (training) load can correspond to different internal loads between and within individuals at different times [22, 23]. This was one of the main reasons for the classification of training load into external and internal load: to conceptually differentiate the quantification of the activities performed by athletes (equivalent to the external dose) from the internal psychophysiological responses induced by these activities (i.e. internal dose), which are the mediators ultimately determining the adaptations (Box 1).

The central section of Fig. 1 illustrates the additional terminology of proximal and distal causes that is sometimes used to describe the causal relationships between variables. This simple causal structure is also important from a practical perspective because it emphasises the mediating role of the internal (training) load and it highlights that to alter the internal load, we typically need to manipulate the external load. For the remainder of this article, we will use the term “exposure” and “training load” when referring generically to both (1) external dose or load and (2) internal dose or load concepts. We will use the terms external dose/load and internal dose/load as per the aforementioned definitions.

### Box 1. Why not use exposure and dose instead of load?

According to what has been presented in the previous section, the use of the terms “training exposure”, “external training dose” and “internal training dose” (i.e. “exposure” and “dose” instead of “load”) would be reasonable and technically appropriate. However, the term “load” is firmly entrenched within the sports science literature, with the first reports using the term training load to indicate the amount of training presenting back in the 1980s [41–43] and a vast quantity of research utilising the term presenting thereafter. We believe that the continued use of the term “load” in sports science is appropriate as this term communicates the amount of something and concurrently conveys the idea of demand and challenges imposed on the organism (while dose or exposure is more “neutral”). These connotations have contributed to the adoption of “load” in various scientific areas (e.g. allostatic load, cognitive load, toxic load, viral load). While the meaning of the term “load” may vary between contexts, polysemy is common and because of the long-standing use of the term load in sports science, we find it very unlikely that practitioners and even more so sports scientists will confuse the meaning of this term with alternative meanings found in other contexts (e.g. mechanical) [8]. Accordingly, we maintain that keeping and continuing to use “load” instead of “exposure” and “dose” is reasonable. Ultimately, however, it is up to the researchers and the scientific community whether they use “load” or “exposure/dose” as either is acceptable.

## Identifying the Measures of Exposure (External and Internal Dose)

﻿In epidemiology and toxicology, the measurement of the internal dose is not always straightforward and can rely on indirect markers and products (e.g. biomarkers as adducts) [44, 45]. Sometimes, the internal dose may not be available at all and therefore researchers estimate the internal dose from its relationship with the external dose [39]. Similar challenges (on what direct or indirect marker to select) occur in sports science and medicine. Every exposure includes multiple components, where different components may affect different outcomes. If we are interested in understanding how manipulating an exposure will affect a specific outcome (i.e. within a causal context), the measure of exposure (either external or internal) should reflect, directly or indirectly, the component of the exposure that causes (i.e. the mechanism or mediator of) that particular outcome of interest [24, 36]. For example, let us assume a sports researcher (or practitioner) is interested in understanding how to manipulate small-sided games to obtain improvements in sprinting (hypothesised to be one of the determinants of match performance). The training load of small-sided games can be quantified in a variety of ways including measures of external load (e.g. time or distance covered running at various speeds, number of accelerations) and internal load (e.g. heart rate, lactate or perceived exertion). Therefore, if it is considered that the neuromuscular stimulus provided by the small-sided games is the mediating mechanism that improves sprinting ability, we should use a measure of exposure that reflects this neuromuscular stimulus. However, the presented examples of internal load measures cannot reasonably be expected to reflect the neuromuscular stimulus. Consequently, a measure of external load may be used as a substitution (surrogate) of the internal mediating mechanism (neuromuscular stimulus). For example, if it is hypothesised that running above a certain percentage of maximal speed provides an adequate reflection of the neuromuscular stimulus mediating the response of interest, this external load measure can be selected. Similarly, if it is anticipated that the accelerations completed in a certain range adequately reflect the neuromuscular stimulus, this measure can be selected as an alternative (or additional) measure. Ideally, the appropriateness of a measure of external training load as a reflection of the neuromuscular stimulus should be supported by evidence from studies. In scenarios where a lack of research exists, the selection of a measure should at least be based on a well-thought-out hypothesis linking the measure to the mechanistic stimulus. Examining the extent to which measures of training load reflect the components causing the mediating mechanisms of an outcome is the goal of the validation process.

In a practical setting, mechanisms reflected by new measures or metrics are often inferred by their label. Unfortunately, many labels or proprietary metrics appear to be developed and selected primarily to distinguish companies’ products from others and serve marketing purposes rather than being mechanistically motivated and validated (see Sect. 7 on the validation process).

To summarise, in sport and exercise, if a measure of training load (external and internal load) is to be used for the purpose of optimising training, this measure should be reflective of the mediating mechanisms through which the specific physical training (factor/agent) is anticipated to cause the effects/responses of interest, i.e. changes in sporting performance or health-related outcomes (e.g. athletic injuries). We again highlight that the term "optimise”, very commonly used in sports science, implies the active manipulation of the external load to generate a specific internal load that ultimately influences (in a positive way) the outcome. Accordingly, optimisation implies causation, and therefore a causal exposure-outcome framework (known or hypothesised) is needed to guide the identification of the measure of exposure [24, 33]. Basic (e.g. physiological) research and frameworks are useful in guiding our understandings of potential mediating mechanisms and the corresponding measures. An example of applying this process to training load and hypothesised mechanisms of injury is provided in two recent papers [34, 46]. These frameworks can also be utilised to inform and develop causal directed acyclic graphs [47], which can then be used to guide appropriate statistical analyses. Importantly, these frameworks are proposals and revising or replacing frameworks when updated knowledge is presented is a normal step in the scientific process. Regardless, frameworks have an important role in presenting causal assumptions transparently and avoiding or limiting ad hoc and post hoc explanations, thus reducing bias and cherry picking.

## Metrics

Once an appropriate measure of exposure is identified, we need to determine the relevant dimensions (metrics) required for the quantification [36]. There are a variety of possible operationalisations leading to different metrics and the choice depends on the exposure/dose–response process of interest [48]. Typical (static) metrics in epidemiology are average, peak, duration and cumulative exposure (CE) [40, 48]. In this section, we elaborate on CE because similar approaches are commonly used within sports science and medicine. Within this metric, exposure (external and internal dose) is usually operationalised according to two dimensions: intensity (I) and duration (T) [40]. Duration is the time period during which the amount of a substance (or training, in a sports context) is delivered. Intensity, according to Checkoway et al. [40] “represents the magnitude of the amount of a substance that potentially can enter the body and be delivered to the biological target(s).” It can also be measured as a rate, i.e. “the rate at which a substance is brought in contact with the body” [49]. Mathematically, the rate is simply the slope of the magnitude versus duration relationship, which may vary over time depending on the context.

Cumulative exposure is calculated as CE = I (intensity) × T (duration), or more generically, if intensity varies over time, as a ﻿time integral of exposure intensity depicted in Eq. 1:

1 $${\text{CE}} = \mathop \smallint \limits_{t1}^{t2} I\left( t \right) {\text{d}}t,$$

where the duration is the interval (*t*_1_, *t*_2_), *I* is the intensity and *t* is the time. The formula for the cumulative dose is obtained by substituting *I* with *I *_*D*_ (dose intensity, where dose refers to internal dose) [49].

**Fig. 2:**
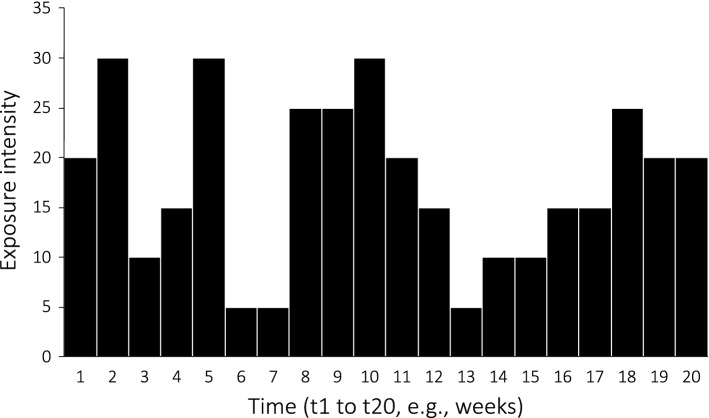
Hypothetical exposure history from the start at, t_1_, to the end, t_20_ (adapted from Checkoway et al. [40]). The sum (∑) of the black rectangles, one for each time interval (from *t*_1_ to *t*_20_) corresponds to the measure of cumulative exposure (simplified representation of integral calculation)

An easier way to present the same method is shown in Eq. 2 [40]:

2 $${\text{CE}} = \mathop \sum \limits_{i = 1}^{N} C_{i} \cdot t_{i} ,$$

where *C* represents the exposure level (i.e. intensity) and *t* corresponds to each time interval as shown in Fig. 2 where the sum (∑) of the black columns, one for each time interval (from t_1_ to t_20_) corresponds to the CE.

This method for CE resembles the way in which training load has historically been quantified. For example, common measures such as training impulse (TRIMP) [50–52] and session-rating of perceived exertion [53] are based on the multiplication of intensity (eventually time weighted) by duration. For the American College of Sports Medicine, the weekly volume formula (for aerobic activities) is the product of frequency, session duration and intensity [54]. In resistance training, volume is sometimes defined as the product of sets and repetitions, or the product of set, repetition and load lifted (absolute or relative) [55–57]. These variations are all reasonable measures of CE and their choice depends on the practical or research goal.

Recent opinion articles have highlighted that combining exercise intensity and duration to form a single cumulative metric has limitations [7, 58, 59]. Many of these limitations have also already been acknowledged in epidemiology [60]. To elaborate, this approach provides an estimate that represents the average effect across all individuals with the same CE, and does not take into account the pattern of exposure over time. As a practical example, consider that the exposure occurs over time as it does in smoking. Using a simplified summary measure of the dose over time (e.g. pack-years of smoking) or any cumulative dose over a recent period, we obtain an average estimate across individuals who smoke 1 pack/day for 1 year and individuals who smoke half a pack/day for 2 years [58]. If we are interested in the differences between these two groups, then such a simplification is not justified and we might consider using intensity, duration *and* their interaction as separate variables in one statistical model [59, 60]. More complicated questions might require biological models of (disease) processes that are dynamic in nature (time varying) and account for the response of biological systems to changes in exposure and internal conditions (for more details see *Chapter 16* in Smith and Kriebel [61]).

It follows that the cumulative measures of exposure and any other metrics used in epidemiology and in sport and exercise science or medicine cannot be considered right or wrong per se, as their validity and appropriateness depend on specific details such as the purpose, what aspects of the exposure the practitioners or researchers are interested in, the acceptability of the limitations of the metric, and whether the assumptions on which the metric are based are reasonable and eventually testable. There are instances where the simple duration of the exposure is the only variable at one’s disposal, and while limited, this information may still be appropriate and meaningful in many contexts [62].

## Dose–Response

Dose–response (sometimes referred to as exposure–response depending on the context) is another important concept common in epidemiology that describes the relationship between the amounts of a factor/agent (i.e. dose) and the responses (e.g. risk of an outcome). This relationship can be monotonic, which in an epidemiological context means the risk of a disease always increases as the intensity or duration of exposure increases [63], or it can exhibit other patterns [64]. In sport, a well-known theoretical dose–response function is the parabola (or “inverted U” shape). This function is based on the concept that performance improves with increases in training load up until a maximum level is reached (vertex), and beyond this point, further increases in training load result in a deterioration of performance (overreaching and overtraining) [65]. Other more complex methods of modelling the cumulative effects of time-dependent exposures (such as training load) on continuous and binary outcomes have been proposed in epidemiology, and these models can also be applied to sport [64, 66, 67].

### Defining the Response (Outcome) of Interest: Surrogate and Intermediate Outcomes

In sport and exercise science or medicine, there are two main responses of interest: performance and health-related outcomes [23]. When measures of these two main responses are unavailable, surrogate outcomes or intermediate responses that are considered to be determinants of performance or health-related outcomes may be utilised. For example, we may be interested in the effect of aerobic training (or any other intervention) on cycling performance, and we use a simulated time trial as a surrogate of actual on-road performance. Alternatively, we may want to examine the relationship between an appropriate measure of resistance training (external or internal dose/load) and strength under the assumption that a higher level of strength affects (i.e. mediates) sporting performance or health-related risks (outcome/responses of interest). In this case, strength is a mediator of the effect of the exposure (resistance training) on performance or health and can be used as an intermediate outcome.

A surrogate *outcome* is especially useful when primary outcomes (commonly termed “endpoint” in clinical research) are difficult to measure or require a very long follow-up, impacting both the feasibility and costs of studies, as well as utility in a practical environment [68–72]. This is also applicable to sports science and medicine, as actual performance or health-related endpoints are often difficult to measure, in both research and applied settings. However, it is important to distinguish between surrogate and intermediate outcomes [73] because their interpretation and implications differ.

**Fig. 3:**
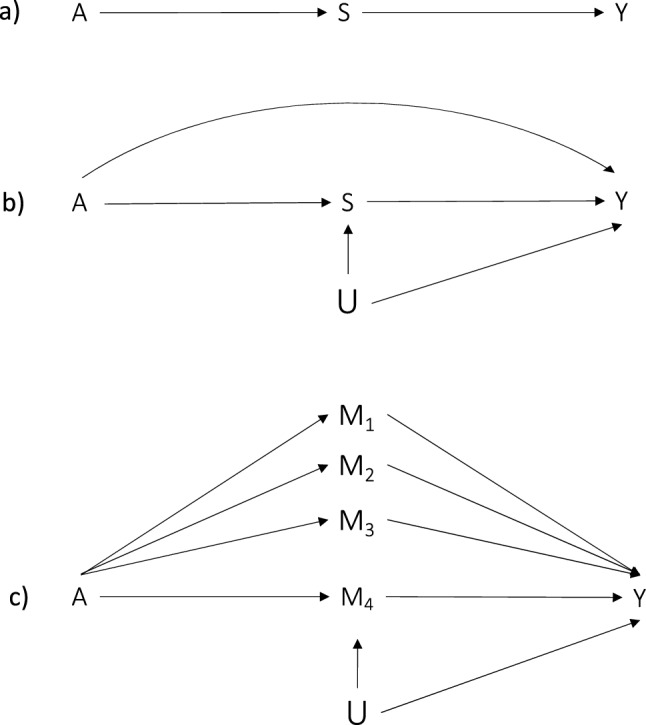
Directed acyclic graph representing **a** surrogate outcome (S) fully mediating the effect of A (e.g. training) on the response of interest, Y (e.g. performance), **b** surrogate outcome (S) with an unmeasured confounder (U) of the S-Y path and another causal path between A and Y, independent from S, and **c** hypothetical additional mediators (M_1_ to M_3_) of the A-Y causal path, where the surrogate outcome is actually just another mediator (M_4_) that can be used as an intermediate outcome

Consider a surrogate outcome (S) for the primary outcome Y (e.g. performance). By knowing the effect of the exposure A (e.g. training) on S, we can predict the effect of A on Y, which is the response of interest [68–70]. To be considered a strong surrogate outcome (i.e. as *substitution* of the primary outcome), S should *fully* (or *mostly*) mediate Y [71]. This is shown by the causal directed acyclic graph (DAG) presented in Fig. 3a, where S is the only mediator in the causal path. However, although a scenario where S fully mediates Y is ideal, it is rare.

A more realistic causal DAG is represented in Fig. 3b, where there are unmeasured confounders (U) of the relationship between S and Y and another causal path may exist between A and Y, independent from S. This other path can be mediated by other mechanisms (M_1_ to M_3_) as shown in DAG 3c, where S is just one of various mediators used as intermediate outcomes (M_4_) and not a surrogate outcome. The DAGs in Fig. 3 are simplified scenarios presented for educational purposes. The causal structures in studies are often much more complicated and can include additional confounders (common causes) and colliders (common effects). Finally, although causal DAGs are helpful to illustrate some assumptions, they do not commonly illustrate effect modification or interactions (see the ESM for graphical diagrams using sufficient causal sets that do illustrate interactions).

In a scenario such as causal DAG 3c with an unmeasured common cause of the mediator and the outcome, if we measure only the effect of A on the intermediate outcome (M_4_) and not the other mediators (M_1_ to M_3_), no accurate inferences can be made about the magnitude of the overall effect of A on Y [74]. To emphasise the importance of accounting for all mediators, scenarios such as presented in DAG 3c can lead to the surrogate paradox [68, 75]. This paradox occurs when other mediators in addition to S (M_4_ in DAG 3c) exist (S is incorrectly identified as a surrogate outcome when it is actually an intermediate outcome) and the effects of the other mediators influencing Y are in the opposite direction compared to the effect of S on Y. For example, the effect of A on S (M_4_) may increase Y, but the effect of A on M_1_/M_2_/M_3_ may decrease Y. If we incorrectly considered S to be an appropriate surrogate for Y, we would conclude A is beneficial when the true overall effect of A on Y is harmful, resulting in a paradox (false-positive result) [68, 70, 75]. It follows that a surrogate outcome should be *consistent*, meaning that a change in a particular direction of S should always reflect a directionally consistent change in the primary outcome (Y) for the specific context described [68]. To illustrate this with an applied sporting example, a practitioner or researcher can find that an exposure has a positive effect on strength, which is assumed to be the main mediator of a given improvement in performance outcome. However, the actual measured effect of A on performance outcome suggests A negatively affects performance. If we know, however, that strength is only one of the many mediators, there will not be expectations regarding the direction of the changes in the performance outcome; i.e. we are simply examining the effects of training on one mediator. Understanding or hypothesising the role of the measures of exposure and response (outcome) in the causal pathway drastically changes their interpretation.

To further clarify using the aforementioned examples in sport indicates that we need studies showing that the time trial (surrogate outcome) is predictive of the actual (on-road) performance of a cyclist or that strength (mediator used as intermediate outcome) is causally related to the real performance of interest. However, in sports and exercise, these assumptions are commonly speculative, and some evidence is needed to support them.

## Validity of the Measures of Exposure or Dose

As explained in Sect. 4, within a causal context (i.e. when we want to manipulate the exposure to alter the outcome), the measure of exposure should reflect a component that is the mediating mechanism for the exposure-outcome causal path. Consider the question posed in Sect. 4: Does a measure of the time spent running above a given percentage of maximal speed during small-sided games provide an appropriate reflection of the neuromuscular stimulus experienced to improve sprint performance? There are several challenges to consider.

First, our external dose is a measure of running time above a speed threshold (measured), our internal dose is the neuromuscular stimulus (unmeasured) and our intermediate outcome of interest is sprint performance (measured), under the assumption (unverified) that sprint performance is a determinant of match performance (unmeasured). If we want to make inferences about the effect of neuromuscular stimulus on sprint performance, we can think of the running time (external dose) measure as a “surrogate” for internal dose [33]. This must be justified with empirical evidence or at least a coherent theoretical framework.

If an observational study with no biases finds that our measure of running time above a certain threshold is associated with improved performance, there remain two possibilities. Either the exposure does provide a neuromuscular stimulus that improves performance (with or without effects on performance that are independent of a neuromuscular stimulus) or the exposure causes a change in performance only through a mechanism that is independent of a neuromuscular stimulus (e.g. improvement of the skills). In other words, the selected measure of exposure may appropriately reflect an identified mechanism of interest; however, this mechanism is irrelevant (or marginally relevant) to the outcome. In short, this measure of exposure may be a valid reflection of an incorrectly identified mechanism.

To summarise, the validation process within a causal context includes two steps. The first step consists of providing evidence that the selected measure is a valid reflection of the putative causal mechanism linked to the outcome, i.e. evidence about its construct validity where the construct (latent variable) is the hypothesised mechanism. The second step requires the examination of whether this putative mechanism is actually causally related to the outcome of interest. From a practical perspective, to complete this process, a measure of the mechanism is needed, which is the measure validated in the first step. This measure can then be used in a purposely designed study to examine the causal role of the proposed mechanisms (mediators) on the outcome of interest.

Finally, we want to highlight that validity is *not* an absolute concept and is context dependent [76, 77]. For example, heart rate (or VO_2_) can only be used to quantify one aspect of training load, the stimulation of “one part” of the cardiorespiratory system. Accordingly, heart rate (or VO_2_) is only valid for the cardiorespiratory component of the training load related to its specific mechanistic effects and cannot be expected to reflect other relevant mechanisms pertinent to athletic training (such as the stimulation of the anaerobic or neuromuscular system). Therefore, heart rate (or VO_2_) is a valid measure of exposure for certain questions related to the cardiorespiratory component of the training load but would be an invalid measure of exposure for questions related to muscle damage or the neuromuscular stimulus experienced. In conclusion, understanding “validity for what purpose” and the context is essential for a correct interpretation.

### Dose–Response as Evidence of Validity

As a valid surrogate for our question requires particular causal relationships, appropriate causal inference methods [78–80] are needed to examine whether the dose–response relationship is indeed causal [79, 81]. Some investigators infer that a measure is a valid surrogate (of the internal dose or outcome) if there is a dose–response relationship (biological gradient) because it is one of the Bradford Hill criteria of causality. However, associations between the dose of exposures and responses may be due to confounding and not causality [28, 82, 83], or multiple independent effects of the exposure.

Therefore, at the forefront, the well-known differentiation between causal and non-causal (e.g. predictive) relationships [79, 84–86] becomes relevant when interpreting the dose–response as evidence of validity. If the relationship is not causal, we can still use the dose–response relationship to explore or to predict. For exploration, the relationship can be utilised to develop hypotheses. For prediction, the validity only concerns the ability to accurately (or acceptably) predict the likelihood of an outcome or future event (forecasting), and the prediction model cannot automatically be considered causal or reflective of causal factors [79, 84, 85]. In our previous example, our measure of small-sided games running time above a given threshold might have had a dose–response relationship with a neuromuscular stimulus and with performance even though a neuromuscular stimulus did not mediate the effect. If true, developing new interventions to change the neuromuscular status would not improve performance. This suggests that it is not appropriate to believe that manipulating a measure of exposure will change the outcome simply because it is one of the features of a prediction model; this hypothesis would still have to be tested empirically.

Similarly, the absence of a dose–response relationship may occur for reasons other than the non-validity of the measure of exposure or the non-causality of the mechanism (i.e. causation is still possible). For example, the identification of a dose–response relationship can be more difficult when a substantial effect occurs above a threshold (of exposure) or when there is a ceiling effect in the response. While a causally potent biological aetiology is certainly desirable, and detailed causal knowledge provides arguably the strongest foundations for the validation process, such an approach has many challenges. For example, individual causal factors are likely to function and interact in complex ways, and their causal roles are sometimes best understood in terms of the larger complex system in which they are embedded. This is especially true if one is interested in providing “personalised” training programmes. For example, consider that an outcome only occurs if factors A and B are present together. An intervention that sets A = 1 will have a positive effect if the athlete has factor B, but no effect if the athlete does not have factor B. Further, various mechanisms may act simultaneously and interact to elicit a response of interest [87].

## Conclusions

Sport and exercise science, and medicine have adopted many terms from other scientific fields. By highlighting the parallels between them, we have illustrated how the concepts of external and internal training load are coherent and consistent with notions from some fields of epidemiology (and pharmacology). Training load is a term reflecting the general concept of exposure, while the separation of training load into external and internal training load closely mimics the separation of exposure into external and internal dose. These subdimensions allow for the differentiation of measures of training-related behaviours from the internal psycho-physiological stimulus (internal dose) induced by these behaviours (i.e. formalisation of the causal relationship between the training load sub-dimensions).

Validity is not an absolute concept; it is context dependent and is derived from a variety of methods and sources that contribute to the research base for and against the theoretical framework supporting a construct. Additionally, the validation process depends on the appropriate selection of the measures of exposure and the response of interest, as per the context. Ideally, a measure should reflect the mechanisms that, at least theoretically, link the exposure to the targeted effects/responses. However, aetiological pathways facilitating sporting performance or health-related outcomes are complex and may be dependent upon a variety of mechanisms acting concurrently and potentially interacting with each other. It is therefore unlikely that a single measure can reflect all the mechanisms mediating the response of interest.

## Practical Applications

The fundamental goals of training are to improve athletic performance, reduce the risk of injury or improve health, sometimes in an “antagonist” way (e.g. performance and injury risk). Any measure of exposure should provide information to support the planning and execution of a training programme aimed to improve characteristics that are causally linked to performance (or to any outcome of interest). The take-home message of this article is that the measures of training load (to support and optimise the training process) should be chosen wisely based on a plausible relationship with a mechanism of interest and the evidence supporting it. Practitioners may hopefully use this information to aid with interpreting and adapting the training–load relationship into practice, as there is often a disconnect between the day-to-day use of training load (as a measure of exposure that can be modified to causally influence an outcome of interest) and how its dose–response relationship is often investigated in scientific articles (largely exploratory and descriptive).

A recent phenomenon of concern is the development, introduction and adoption of several new metrics that lack conceptual support: i.e. there is no explicit theoretical framework that reasonably links these metrics to the mechanism that it is supposed to reflect, nor whether these mechanisms are reasonably related to the outcome of interest (e.g. performance enhancement or better health). In other words, these metrics are not theory driven, and the “burden of proof” has been reversed; it appears that measures of exposure are presented, and it is then left to others to try and understand what it actually measures and whether it can be useful. It does not matter how sophisticated or “advanced” a metric appears. If it cannot be connected to a plausible mechanism (or relevant responses), it is likely of little use to support and optimise the training process in practice. However, such metrics can still be used for exploration and to generate hypotheses from a research perspective.

Finally, we would like to emphasise that with this and previous articles [8, 9, 22, 23, 88] we are not claiming that the provided classification and framework *must* be used; we have simply presented several arguments and “informal” conceptual analyses to explain why we believe this conceptualisation of training load and its components can be useful to scientifically investigate the physical training process.

## Supplementary Information

Below is the link to the electronic supplementary material.

Supplementary file1 (DOCX 143 KB)
