# Supplementary material for: Understanding Training Load as Exposure and Dose
Source: Sports Med. 2023 Apr 6;53(9):1667–79. doi: 10.1007/s40279-023-01833-0 (PMC10432367; doi:10.1007/s40279-023-01833-0)
Supplement: Supplementary file 1 — Supplementary file1 (DOCX 143 KB) [file 40279_2023_1833_MOESM1_ESM.docx]

# **Supplementary Material**

## Dynamic versus Static Systems:

The sport and exercise medicine community has started to consider both causal diagrams^1^ and dynamic or complex systems^2-4^ in their analysis and conceptualization. In our article, a static system perspective was adopted. However, static does *not* mean that a system does not change over time, nor does it mean that the data are not generated by a dynamic system (see next section). A static system “simply” assumes time-invariance within a discrete time interval (point in time). While the use of static systems may appear to be an “over-simplification”, their use in clinical research is useful. Indeed, their adoption and application have facilitated the successful identification and confirmation of causal paths that have resulted in effective treatments.^5^ This does not negate the value of a dynamic system approach but simply highlights that there may be different (methodological and philosophical) approaches to answering similar research questions. Both approaches can produce findings that are useful in a real-world setting and can help to generate knowledge. How to reconcile the two approaches is an area still under investigation and debate.^6-8^

There are mathematical and philosophical differences between these two different perspectives and these differences affect how we simulate and analyze data. While exploring these differences and their implications in detail is beyond the scope of this paper, one important distinction worth highlighting is that these frameworks often utilise the same words with different meanings, which can lead to confusion. The main purpose of our article was to explain concepts related to exposure and dose, and not to address the different philosophical approaches to causation. During the review process, however, a discussion regarding the use of static vs dynamic systems perspectives arose, which may be of interest to others. Therefore, we elaborated on the comparison in this supplementary material. This can be useful for other readers embracing a dynamic system perspective and may not be familiar with causal inference and counterfactual framework principles, terminology and methodology. We, therefore, provide a brief overview of three important concepts below: (1) “bidirectionality” (or circular causality) versus time-varying exposures, (2) causes versus interactions and (3) the sufficient cause model which shows the multifactorial nature of causation.

## “Bidirectionality” versus “Time-varying exposures”

Conceptually, a dynamic system can be thought of as a system where the value of variables is constantly changing over time but the system is in equilibrium and self-regulates to reach stability.^2^ Physiologically, homeostasis is a state of equilibrium. Mathematically, equilibrium means the average value remains constant over the chosen period of time. In other words, sampling data at random time points is always expected to give the same average value. Consider that blood pressure is in equilibrium (e.g., controlled by the carotid sinus) and constantly fluctuating around an average value. Measuring blood pressure at any time point will on average, give the same value as blood pressure at another time point. When one intervenes on a variable and “changes it” (e.g., medication), the system will achieve a new “equilibrium” value (e.g., mean blood pressure). Because the values of variables in dynamic models are constantly changing even if in equilibrium, and there may be multiple factors that affect each other over time, the language and graphical models used to describe dynamic systems often refer to circular causality (“bidirectionality”).^9^ For example, alcohol might cause smoking, and smoking might cause alcohol consumption (Figure 1S-A).

In a static system (e.g., structural causal models, causal directed acyclic graphs (DAGs)), a variable is defined both by its construct and the *time* at which it is measured. As the value of a variable may change over time, such variables are known as time-varying variables or exposures. Using the example of alcohol and smoking, alcohol consumption on Jan 1 can cause smoking on Feb 1, but alcohol consumption on Feb 1 cannot cause smoking on Jan 1. Therefore, from the causal inference perspective, causal DAGs depicting the causal relationships between alcohol consumption and smoking would be represented by one node for alcohol consumption at each time point, and one node for smoking at each time point, i.e., alcohol consumption at one point in time is a different variable to alcohol consumption at another point in time, with the same applying to smoking. To elaborate, when constructing our DAG (Figure 1S-B), we might include an arrow from (1) alcohol Jan 1 to smoking Feb 1, (2) alcohol Jan 1 to alcohol Feb 1, (3) smoking Jan 1 to alcohol Feb 1 and (4) smoking Jan 1 to smoking Feb 1. However, we would not include arrows from alcohol Feb 1 to smoking Jan 1, or from smoking Feb 1 to alcohol Jan 1, as it is not possible for something in the future to cause something in the past. Conceptually, the static model can be expanded (by using separate nodes for each timing) so that each timepoint is only one minute or one second apart, essentially providing the causal relationships in the dynamic model (but remaining acyclic, i.e., no variable can be the cause of itself). Comparing the dynamic to static models, it becomes obvious that the “bidirectionality” language used in dynamic models is possible because cause-effect temporality (i.e., time) is treated differently (e.g., cyclic diagrams, Figure 1S-A) than in static systems (e.g., acyclic diagrams, Figure 1S-B and 1S-C).

**Figure 1S.** Bi-directionality causality represented in dynamic systems (A) and in static causal directed acyclic graphs (B and C). For simplicity, the diagrams incorporate just two variables, smoking and alcohol, but the same applies to more complex causal paths. Figure 1S-A represents causal loop diagram^10,11^ with causal circularity (bidirectionality) when time is only implied and not explicitly included (only time delays are normally included in this kind of graphical representation). The internal loop or the signs – and + normally representing positive (reinforcing) or negative (balancing) effects presented given the generic examples. Figure 1S-B and 1S-C represent causal directed acyclic graphs for the same data generating process, where each variable is defined by its construct (smoking, alcohol) and the time it is measured. In (B), there is no intervention. In (C), there is an intervention setting Smoking_Feb 1_ to a specific value for different participants: do(Smoking_Feb 1_) (modified from^12^). Causal directed acyclic graphs can also include positive and negative effects but are not routinely used.^13^

## Causes and Interactions

In dynamic systems, cause is *sometimes* restricted to a system state (which is the cause of the next state), or the self-organising process *causing* the emergence of (new) properties in complex systems..^14-16^ However, cause is also often used more broadly, and consistent with the use of the term in static systems.^15,17,18^

In static systems, a cause can be a single variable, and this variable can interact with others. Restricting the word “cause” to a state and self-organization, while coherent with some specific views of causation from a dynamic system perspective,^14^ is inconsistent with contemporary uses of the word in most of science and medicine, on which our article was based. To elaborate, consider a randomized trial of codeine for pain control. The mean pain level in the group after receiving codeine is 4 and the mean pain level in the group after receiving the control is 8. The standard conclusion is that codeine causes pain relief. However, codeine must first be converted to morphine by the liver to relieve pain. This enzyme is missing in ~10% of the population. Therefore, using the proposed dynamic systems interpretation of cause above, we could not say codeine is a cause of pain relief. Rather, we would only be able to say the interaction between codeine and a specific enzyme (and other system components such as the ability to absorb codeine, enzymes that breakdown codeine) was the cause of the pain relief.

In dynamic systems, the same concepts occur but the terminology is different. Consider the context of describing how a system changes in response to an external intervention perturbating its dynamic.^19^ The external intervention can be seen as the intervening variable causing the system to change. Within our article, exposure is the intervening variable, i.e., the variable causing a system to respond to this perturbation (under the dynamic system perspective). Even though new properties of the system emerge as a consequence of the interactions between the different components, feedback loops and through self-organization, this is still compatible with the interpretation of exposure as a *cause*.

## The sufficient cause model

Static models can incorporate the same concept of interactions but under the rubric sufficient component causal model framework.^20-22^ This framework conceptualises a cause as an “act or event, or a state of nature which initiates or permits, alone or in conjunction with other causes, a sequence of events resulting in an effect”.^20^ In this framework, all of the individual causes together are called a sufficient cause, and each of the individual causes that is not sufficient to cause the event by itself is called a component cause. There may be several different sufficient causal sets for an event. When a component cause is present in every sufficient cause, it is called a necessary cause.^20^ One of the acknowledged strengths of this model (compatible with causal inference principles, counterfactual terminology and models) is that it makes clear the multifactorial nature of causation.^21^

Using codeine and pain relief as a concrete example, the component causes a) codeine, b) the converting enzyme and c) any other required factor for the event to occur, together form a sufficient causal set. In the simple causal DAG that most readers may be familiar with, both codeine and the enzyme would be represented by their own nodes, and each would have an arrow to pain relief (Figure 2S-A). The fact that both are required together for pain relief is not indicated on the graph. Although this appears a limitation, it is by design and is required for many of the conclusions we make using causal DAGs.

Although the commonly used causal DAG does not show interactions, sufficient component causes that include mapping out these interactions explicitly can be represented in causal DAGs (Figure 2S-B). These models can also indicate that a cause may be the absence of a variable rather than its presence. For example, the development of phenylketonuria requires both ingestion of phenylalanine and absence of the phenylalanine hydroxylase enzyme.^23^ For a more complete understanding of the strengths and limitations of sufficient component causal DAGs, and solutions to model ﻿to more complicated multicausal relationships, see.^24-27^

**Figure 2S.** (A) Common direct acyclic graph for pain relief using codeine and acetylsalicylic acid (ASA) as the only two causes for simplicity. Both codeine and ASA require absorption, and codeine must be converted to morphine by an enzyme for it to be effective. In B, the required interactions between variables for codeine to be effective, and for ASA to be effective, are indicated within the ellipse of sufficient causes (adapted from^25,26^).

**References**

1. Shrier I, Platt RW. Reducing bias through directed acyclic graphs. *BMC Med Res Methodol.* 2008;8:70.

2. Pol R, Hristovski R, Medina D, Balague N. From microscopic to macroscopic sports injuries. Applying the complex dynamic systems approach to sports medicine: a narrative review. *Br J Sports Med.* 2019;53(19):1214.

3. Stovitz SD, Shrier I. Causal inference for clinicians. *BMJ Evid Based Med.* 2019;24(3):109-112.

4. Stern BD, Hegedus EJ, Lai YC. Injury prediction as a non-linear system. *Phys Ther Sport.* 2020;41:43-48.

5. Pearce N, Greenland S. On the evolution of concepts of causal and preventive interdependence in epidemiology in the late 20(th) century. *Eur J Epidemiol.* 2022:1-6.

6. Bollt EM, Sun J, Runge J. Introduction to focus issue: causation inference and information flow in dynamical systems: theory and applications. *Chaos.* 2018;28(7):075201.

7. Ding Y, Toulis P. Dynamical systems theory for causal inference with application to synthetic control methods. Paper presented at: International Conference on Artificial Intelligence and Statistics2020.

8. Paluš M, Krakovská A, Jakubík J, Chvosteková M. Causality, dynamical systems and the arrow of time. *Chaos.* 2018;28(7):075307.

9. Thomas R. Circular causality. *Syst Biol.* 2006;153(4):140-153.

10. Lin G, Palopoli M, Dadwal V. From causal loop diagrams to system dynamics models in a data-rich ecosystem. In: *Leveraging data science for global health.* Springer, Cham; 2020:77-98.

11. Salmon PM, Stanton NA, Walker GH, et al. Causal Loop Diagrams (CLDs). In: *Handbook of Systems Thinking Methods.* CRC Press; 2022:157-180.

12. Gische C, West SG, Voelkle MC. Forecasting causal effects of interventions versus predicting future outcomes. *Struct Equ Modeling.* 2021;28(3):475-492.

13. VanderWeele TJ, Hernán MA. Results on differential and dependent measurement error of the exposure and the outcome using signed directed acyclic graphs. *Am J Epidemiol.* 2012;175(12):1303-1310.

14. van Geert PLC. Dynamic systems, process and development. *Human Development.* 2019;63(3-4):153-179.

15. Fazekas P, Gyenis B, Hofer-Szabó G, Kertész G. A dynamical systems approach to causation. *Synth.* 2021;198(7):6065-6087.

16. De Wolf T, Holvoet T. Emergence Versus Self-Organisation: Different Concepts but Promising When Combined. Paper presented at: Engineering Self-Organising Systems; 2005//, 2005; Berlin, Heidelberg.

17. Bongers S. *Causal modeling & dynamical systems: A new perspective on feedback*, NARCIS; 2022.

18. Kirkwood CW. System dynamics methods. *College of Business Arizona State University USA.* 1998.

19. Peters J, Bauer S, Pfister N. Causal models for dynamical systems. In: *Probabilistic and Causal Inference: The Works of Judea Pearl.*2022:671-690.

20. Rothman KJ. Causes. *Am J Epidemiol.* 1976;104(6):587-592.

21. VanderWeele TJ. Invited commentary: the continuing need for the sufficient cause model today. *Am J Epidemiol.* 2017;185(11):1041-1043.

22. Tyler JV, Thomas SR. General theory for interactions in sufficient cause models with dichotomous exposures. *Ann Stat.* 2012;40(4):2128-2161.

23. van Spronsen FJ, Blau N, Harding C, Burlina A, Longo N, Bosch AM. Phenylketonuria. *Nat Rev Dis Primers.* 2021;7(1):36.

24. VanderWeele TJ, Shrier I. Sufficient cause representation of the four-way decomposition for mediation and interaction. *Epidemiol.* 2016;27(5):e32-33.

25. VanderWeele TJ. A unification of mediation and interaction: a 4-way decomposition. *Epidemiol.* 2014;25(5):749-761.

26. VanderWeele TJ, Robins JM. Directed acyclic graphs, sufficient causes, and the properties of conditioning on a common effect. *Am J Epidemiol.* 2007;166(9):1096-1104.

27. Vanderweele TJ, Vansteelandt S, Robins JM. Marginal structural models for sufficient cause interactions. *Am J Epidemiol.* 2010;171(4):506-514.
